# Supplementary material for: Exploring Propolis as a Sustainable Bio-Preservative Agent to Control Foodborne Pathogens in Vacuum-Packed Cooked Ham
Source: Microorganisms. 2024 Apr 30;12(5):914. doi: 10.3390/microorganisms12050914 (PMC11124515; doi:10.3390/microorganisms12050914)
Supplement: Supplementary file 1 [file microorganisms-12-00914-s001.zip › microorganisms-2942243-supplementary.pdf]

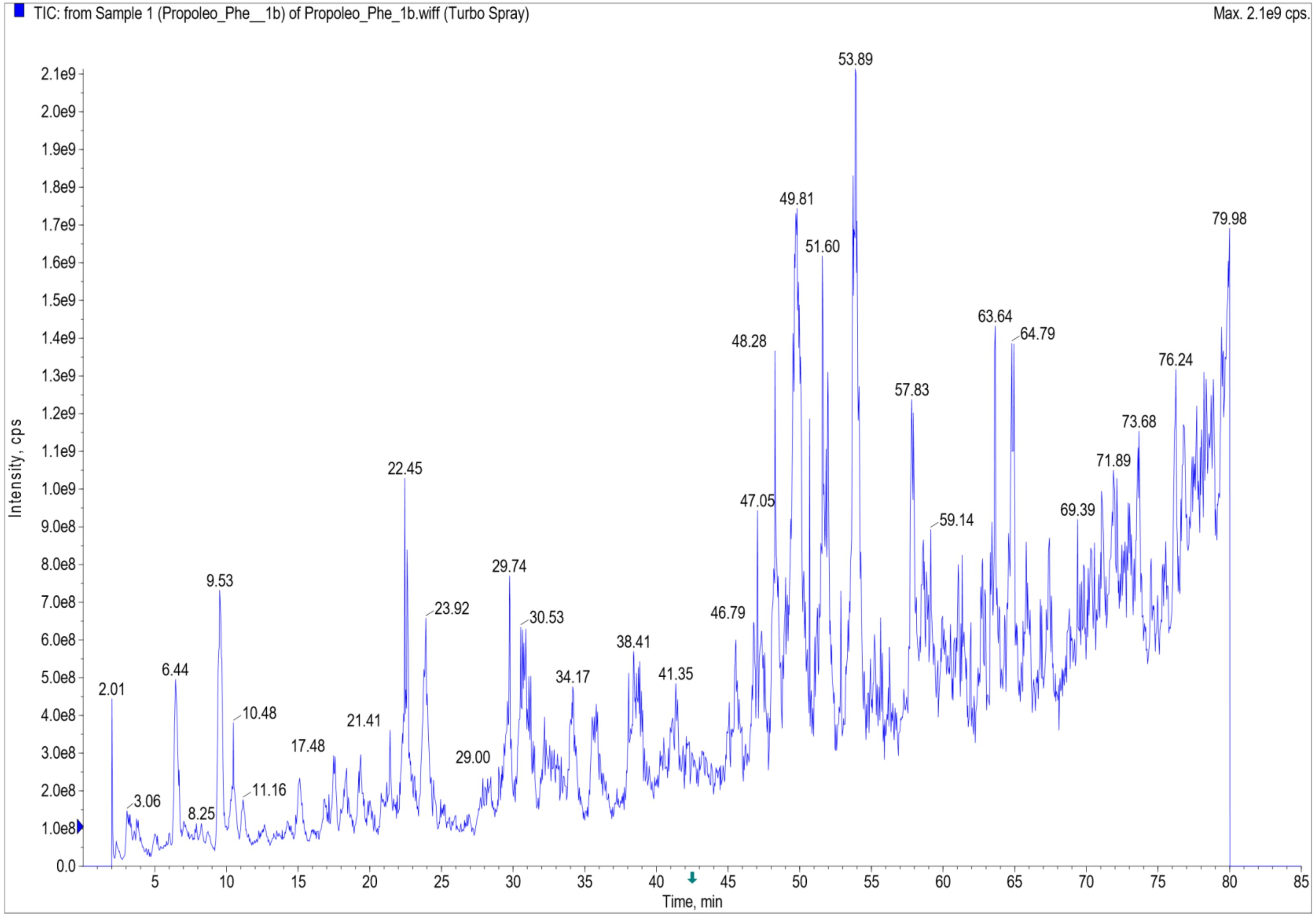

Figure S1. Total Ion Chromatogram (TIC) of the selected propolis.

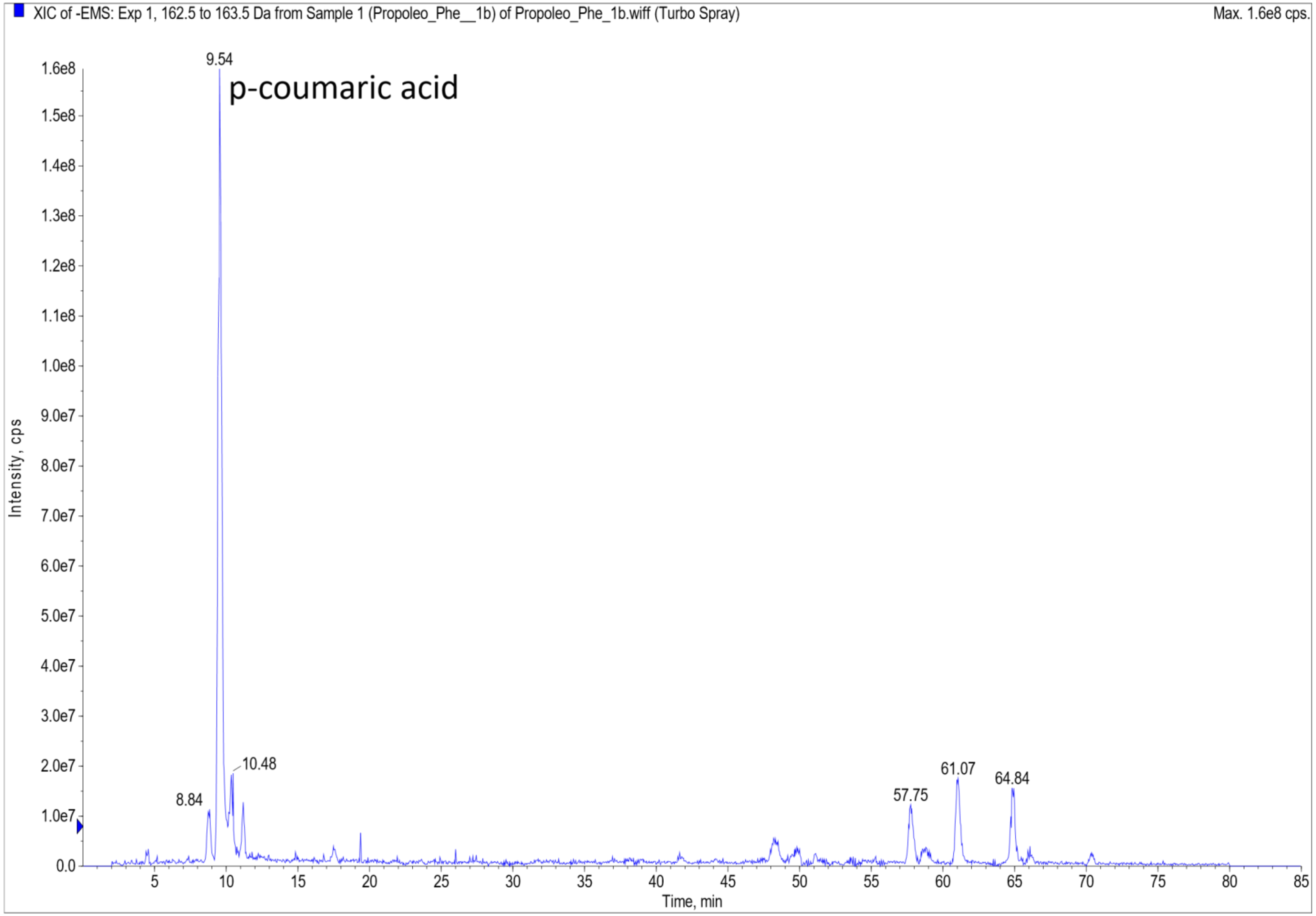

Figure S2. Extracted ion chromatogram of propolis against target mass ions of p- coumaric acid m/z 163

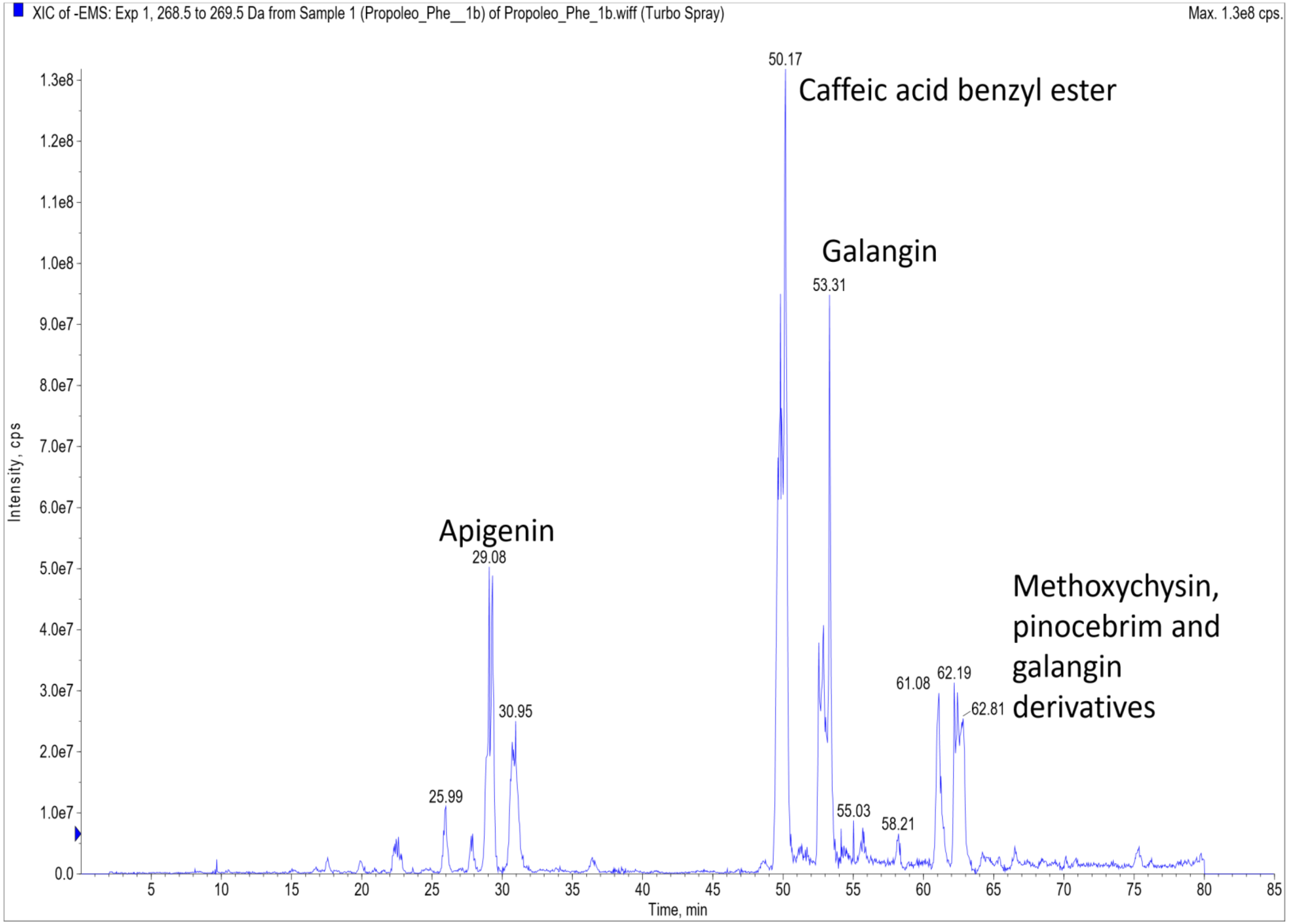

Figure S3. Extracted ion chromatogram of propolis against target mass ions m/z 269

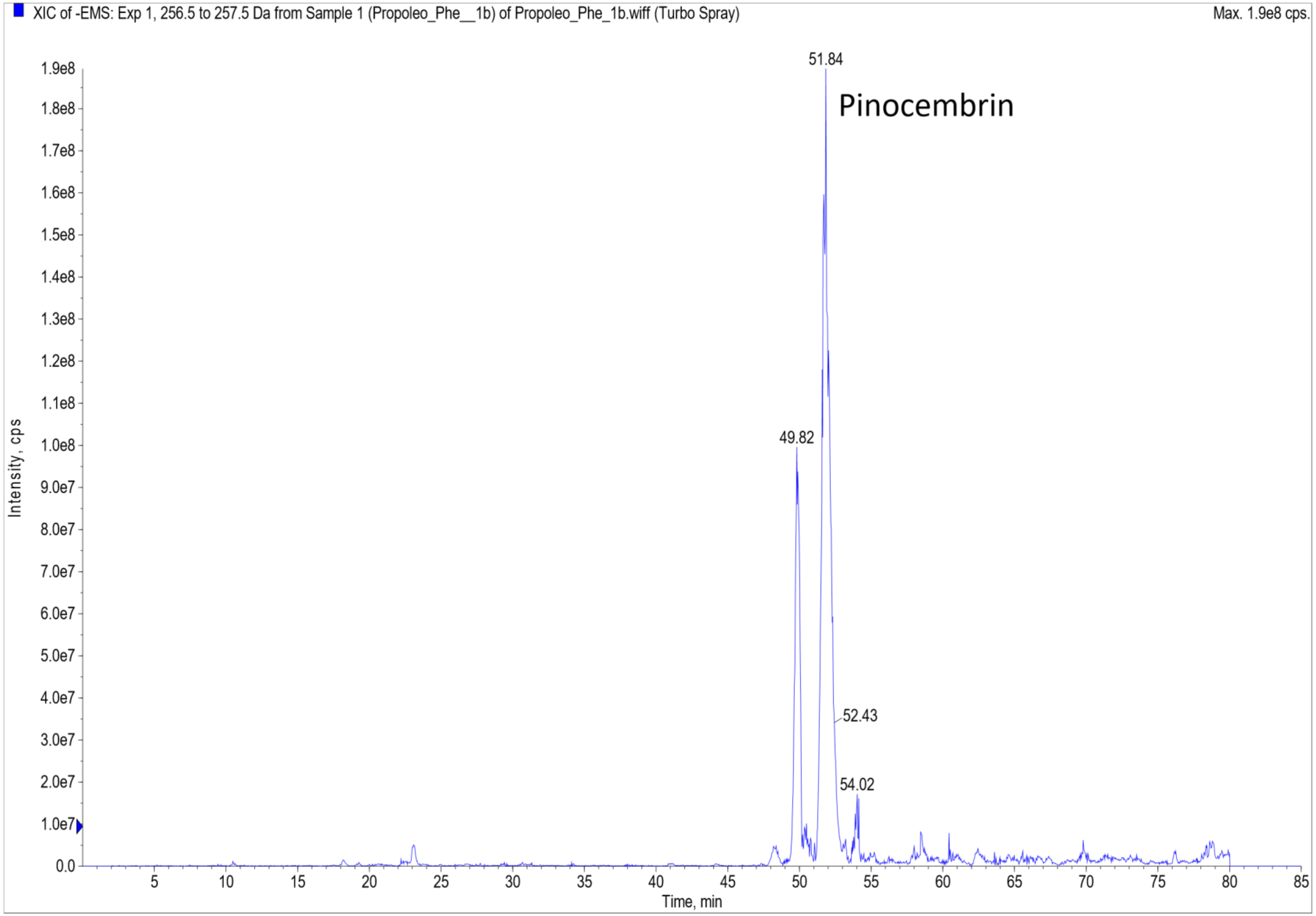

Figure S4. Extracted ion chromatogram of propolis against target mass ions m/z 257
